# Effects of different levels of pistachio (*Pistachia vera*) green hull aqueous extract on performance, intestinal morphology and antioxidant capacity in *Eimeria* challenged broilers

**DOI:** 10.1016/j.psj.2024.103667

**Published:** 2024-03-19

**Authors:** Hadi Noruzi, Fatemeh Aziz-Aliabadi, Zeyad Kamal Imari

**Affiliations:** Department of Animal Science, Faculty of Agriculture, Ferdowsi University of Mashhad, Mashhad, Iran

**Keywords:** antioxidant capacity, broiler, *Eimeria*, intestinal morphology, performance

## Abstract

A total of 576-day-old Ross 308 broilers chicks (male) were used to evaluate the effect of various levels of pistachio green hull aqueous extract (**PHE**) and *Eimeria* challenge on the growth performance, intestinal health and antioxidant capacity. During infection period (25–42 d), treatments included: 1) control + unchallenged (negative control, **NC**), 2) 200 ppm PHE + unchallenged, 3) 300 ppm PHE + unchallenged, 4) 400 ppm PHE + unchallenged, 5) control + challenged (positive control, PC), 6) 200 ppm PHE + challenged, 7) 300 ppm PHE + challenged and 8) 400 ppm PHE + challenged (with 6 replications for each treatment). The outcomes revealed that in the challenged birds, average body weight gain (**ABW**), daily weight gain (**DWG**), and feed conversion ratio (**FCR**) linearly improved with increasing the PHE levels (*P* < 0.05). Infected broilers had lower daily feed intake (**DFI**) compared to unchallenged birds (*P* < 0.05). Villus height (**VH**), villus height to crypt depth (**VH: CD**) ratio and villus surface area (**VSA**) reduced linearly (*P* < 0.05), while muscle layer thickness (**MT**) increased linearly in challenged birds (*P* < 0.05). The consumption of the PHE significantly reduced the excreta oocytes and duodenum and jejunum lesion scores in *Eimeria*-challenged broilers *(P* < 0.05). By increasing the PHE levels, total antioxidant capacity (**TAC**) and superoxide dismutase (**SOD**) levels increased (*P* < 0.05), while the *Eimeria* challenge reduced TAC, **SOD**, and glutathione peroxidase (**GPx**) levels (*P* <0.05). In general, the use of PHE in the broilers diet improved the antioxidant capacity, birds performance, but diminished the excreta oocytes and lesion scores with no negative effect on the intestinal morphology.

## INTRODUCTION

One of the most important illness in the poultry industry is coccidiosis, which is caused by protozoan parasites *Eimeria spp*. It has been stated that this parasite has an adverse effect on the poultry digestive tract and decreased growth performance and increased mortality (Ali et al., 2019). Studies by Lin et al. (2022) and Teng et al. (2021) showed that *Eimeria* mild infection causes a significant decrease in growth performance-related traits. These parasites can reproduce in the mucous epithelium of different parts of the birds’ intestine and cause intestinal inflammation and bleeding, diarrhea, and death (Chapman, 2014). It has been reported that due to the reduced absorptive surface of the brush border in the small intestine, *Eimeria*-challenged broilers have less space to absorb nutrients from the intestinal tract. In addition to shorter villus in the jejunum, reduced nutrient transporters and enzymes in the brush border membrane contributed to reduced amino acid and energy digestibility in coccidiosis (Su et al., 2015; Rochell et al., 2016). Inflammatory reactions have been shown to contribute to the pathology of *E. tenella* infections. Evidence showed that *E. tenella* infection in chickens increased NO concentration, an effect also seen in E. acervulina infection (Allen, 1997). In order to prevent and control coccidiosis, many types of anti-coccidial drugs are applied, but their excessive use has caused concerns due to drug resistance *Eimeria* strains, tissue residue, and increasing breeding costs (Tewari and Maharana, 2011). Therefore, researchers are looking to identify different plant compounds and their by-products and investigate their active compounds, so that they can compensate for the damages caused by this disease (Abbas et al., 2006). These herbal compounds reduce the growth of pathogens through competitive elimination mechanisms and stimulation of the immune system, while they are also effective in increasing the digestibility of nutrients and reducing blood cholesterol (Lee et al., 2003).

Pistachio (*Pistacia vera* L.) a member of the Anacardiaceae family, is native to Asia and generally spread in the Mediterranean area (Ozcelik et al., 2005). One of the by-products of pistachio is its green hull, which makes up more than 60% of pistachio by-products (Arjeh et al., 2020). Pistachio green hull (**PGH**) contains high amounts of bioactive compounds, mainly phenolic compounds (Rafiee et al., 2018). It has been reported that the concentration of dry matter, crude protein, crude fiber, ash, crude fat, and nitrogen-free extract in PGH are 23.00, 11.00, 15.00, 12.00, 6.00, and 55.50%, respectively (Shakerardekani and Molaei, 2020). Various studies have investigated some of the functional attributes of the pistachio green hull extract (**PHE**) such as phenolic materials (Seifzadeh, et al., 2019), antimicrobial (Rajaei et al., 2010) and antioxidant (Abolhasani et al., 2018) activities and its biological profits on human health (Sila et al., 2014). Based on our research, few studies have been done on the effects of pistachio by-products in broilers (Hosseini-Vashan et al., 2020; Ahmadi Kohanali et al., 2022). Considering the widespread cultivation of pistachio in Iran and the easy access to its by-products, as well as taking into account the chemical compounds, the purpose of this study was to investigate the effect of PHE on the growth performance, intestinal morphology and antioxidant capacity of broilers that were infected by *Eimeria*.

## MATERIALS AND METHODS

### Birds, Diets, and Management

A total of 576 one-day-old Ross 308 broiler male chicks were obtained from a local commercial hatchery and reared in pens (1m*1m) for 42 d. Twelve chicks were allocated to each pen. During the starter and grower periods, treatments included 1) control, 2) 200 ppm PHE 3), 300 ppm PHE, and 4) 400 ppm PHE. Twelve replicates were used in each treatment. At the end of the grower phase (25 d), each treatment was divided into two groups (*Eimeria*-challenged and unchallenged broilers). Treatments included 1) control + unchallenged (negative control, NC), 2) 200 ppm PHE + unchallenged, 3) 300 ppm PHE + unchallenged, 4) 400 ppm PHE + unchallenged, 5) control + challenged (positive control, PC), 6) 200 ppm PHE + challenged, 7) 300 ppm PHE + challenged and 8) 400 ppm PHE + challenged. Six replicates were used for each treatment. At d 25 of age, half of the birds were orally gavaged with 20x dose of trivalent live attenuated vaccine (Livacox T, Biopharm Co, Prague, Czech Republic). One dose of vaccine (0.01 mL) contained 300 to 500 sporulated oocysts of each of *Eimeria acervulina, Eimeria maxima*, and *Eimeria tenella*. Before inoculation, the infectious dose of vaccine (0.5 mL) was diluted to 1 mL with distilled water. Control groups were inoculated with 1 mL of distilled water. Ingredients and nutrient composition of diets are shown in Table 1 (NRC, 1994) and diets were formulated to meet Ross 308 requirements (Aviagen, 2022). In the first 3 d of broilers life, rearing place temperature was fixed at 32 °C and then decreased by 3 °C each week to achieve 21 °C and remained stable till the termination of the study. Relative humidity was maintained at 50 to 60% within the rearing period. An 18:6 h light and darkness program was applied during the experiment. Birds had free access to feed and water.

**Table 1:** Ingredients and nutrient composition of the experimental diets (as-fed basis).

Ingredients (%)	Starter (1–10 d)	Grower (11–24 d)	Finisher (25–42 d)
Corn	49.14	53.96	60.75
Soybean meal (44%)	42.23	38.08	32.12
Soybean oil	4.25	4.36	3.84
Limestone	0.97	0.72	0.67
Dicalcium phosphate	2.04	1.61	1.32
Vitamin premix^1^	0.25	0.25	0.25
Mineral premix^2^	0.25	0.25	0.25
L-Lysine HCl	0.07	0.02	0.07
DL-Methionine	0.29	0.24	0.23
L-Threonine	0.01	-	-
Choline	0.05	0.05	0.05
Sodium Bicarbonate	0.11	0.11	0.11
Salt (NaCl)	0.35	0.35	0.34
Nutrient composition (%)			
Metabolizable energy (kcal/kg)	2975	3050	3100
Crude protein	23	21.50	19.50
Lysine	1.32	1.18	1.08
Methionine + Cystine	1.00	0.92	0.86
Threonine	0.88	0.80	0.72
Calcium	0.95	0.75	0.65
Available Phosphorus	0.50	0.42	0.36
Sodium	0.18	0.18	0.18
Potassium	0.99	0.92	0.82
Chlorine	0.26	0.25	0.26

1 Provided the followings per kg of diet: vitamin A (trans-retinyl acetate), 13,000 U; vitamin D3 (cholecalciferol), 5,000 U; vitamin E (D L-α tocopherol acetate), 80 U; vitamin K (menadione), 4 mg; riboflavin, 9 mg; pantothenic acid (D-Ca pantothenate), 25 mg; pyridoxine (pyridoxine-HCl), 5 mg; thiamine, 5 mg; vitamin B12 (cyanocobalamin), 0.02 mg; biotin, 0.35 mg; folic acid, 2.5 mg; nicotinic acid, 70 mg; ethoxyquin (antioxidant), 2.5 mg.

2 Provided the following per kg of diet: Fe, 20 mg; Zn, 120 mg; Mn, 120 mg; Cu, 16 mg; I, 1.25 mg; Se, 0.30 mg.

### Preparation of Pistachio Green Hull Extract

Pistachio green hulls (Ahmadaghaei variety) were obtained from Fakhrabad, Iran. Hulls were dried, ground, and stored at -80C until analysis. Using a liquid-to-solid ratio of 15:1 (at 25 °C for 8 h), one gram of ground green hull was placed in the water, (Rajaei et al., 2010). Folin–Ciocalteu colorimetric method was applied to measure the total phenolic content (Waterhouse, 2002). By a calibration curve obtained with gallic acid, calculations were done. Total phenolic content was represented as milligram of gallic acid equivalents per gram of dry weight. The total tannin was measured by calculating the nontannin phenols and the precipitation of tannins using insoluble polyvinyl pyrrolidone. Difference between total phenol and nontannin phenols showed the total tannins content (Makkar et al., 1995). For standard, gallic acid was applied and the total flavonoid content was measured (Heimler et al., 2005). The dried pistachio green hull powder contained 3931.67 kcal/kg of gross energy, 941.64 g of dry matter/kg, 121.46 g of crude protein/kg, 58.13 g of crude fat/kg, 151.68 g of crude fiber/kg, and 119.83 g of crude ash/kg (AOAC, 2005). The chemical composition of the pistachio green hull extract is shown in Table 2.

**Table 2:** Chemical composition of pistachio green hull extract (**PHE**).

Characteristics	Amount
Total phenol (mg GAE/g DW pistachio hull)	91.32
Total tannin (mg GAE/g DW pistachio hull)	32.03
Flavonoid (mg CE/g DW pistachio hull)	30.46

Abbreviations: GAE, gallic acid equivalents; DW, dry weight; CE, catechin equivalent.

### Growth Indicators

Birds weight from each replicate was determined on 11, 25 and 42 d of age. Growth indicators including average body weight (**ABW**), weight gain (as daily, **DWG**), feed intake (as daily, **DFI**) and feed conversion ratio (**FCR**) were recorded (Noruzi et al., 2023).

### Intestinal Morphology

All stages of the experiment from selection and slaughter of birds and sampling were done according to Noruzi et al. (2023). At the end of the experiment, the parameters including villus height (**VH**), villus width (**VW**), crypt depth (**CD**), and the thickness of muscle layer (**MT**) were recorded. Villus surface area (**VSA**) was measured by (2л)*(VW/2)*(VH). One bird from each replicate was randomly selected and euthanized by cervical dislocation at 42 d. The entire intestinal tract was separated, and 1 cm parts were taken from the mid-point of the jejunum. The sections were fixed in neutral buffered formalin solution (10%) and fixed in paraffin wax later. All histological analyses were done on 5 μm segments, stained with H&E. To assess the morphology of the samples, a computer-connected optical microscope (Olympus model B×51 microscope; magnification 100) was used.

### Lesion Scoring

On d 7 post-*Eimeria* challenge (32 d of age), lesion scoring of the duodenum, jejunum, and cecum was done. One bird per pen was randomly selected and euthanized. The three mentioned parts were assessed according to method described by Johnson and Reid (1970) method with scores ranging from 0 (no gross lesions) to 4 (most severe lesions).

### Number of Oocysts Per Gram of Excreta

The excreta were collected on d 5, 7, 9, and 11 postcoccidiosis challenge. Samples were kept in the refrigerator for measuring of oocysts per gram of excreta. A 10 % (w/v) fecal sample was suspended in a NaCl solution (151 g salt blended into 1 l of water). After shaking, 1 mL of the suspension was combined with 9 mL of a NaCl solution (311 g of NaCl mixed into 1 l of distal water). Then, the suspension was introduced into the McMaster chamber by a micropipette, and the number of oocysts was counted using a microscope (Ali et al., 2019).

### Serum Antioxidant Capacity

On d 42, blood samples were obtained from the same birds that were slaughtered for intestinal morphology evaluation (each bird from each replicate). Assay kits for total antioxidant capacity (**TAC**), superoxide dismutase (**SOD**), glutathione peroxidase (**GPx**), and malondialdehyde (**MDA**) were obtained from Nanjing Jiancheng Bioengineering Institute (Nanjing, China). All of the experiments followed the kit's instructions. The method of ferric reducing-antioxidant power assay was used to measure the TAC (Benzie and Strain, 1996) and detected at 520 nm. The concentration of SOD was determined via the xanthine oxidase method (Winterbourn et al., 1975). The amount of GPx was determined at 412 nm using a spectrophotometer (Hafeman et al., 1974). The MDA level was analyzed with 2-thiobarbituric acid, monitoring the change of absorbance at 532 nm (Placer et al., 1996). The concentration of nitric oxide (**NO)** was determined at 550 nm according to Wang et al. (2008a).

### Statistical Analyses

This trial was performed based on a completely randomized design (**CRD**) during starter (1–10 d) and grower (11–24 d) phases with 4 treatments and 12 replicates for each of them. General Linear Model (**GLM**) procedure of SAS 9.4 (2012) software was used and linear and quadratic contrasts were reported. During the finisher (25–42 d) period, data (growth performance, jejunum morphology, antioxidant activity) was analyzed based on CRD in a factorial test of 4*2 (4, levels of PHE; 2, *Eimeria* challenged and unchallenged broilers) with 6 replications and GLM procedure of SAS 9.4 (2012) software was applied. Kruskal–Wallis test (nonparametric statistical method) followed by the DSCF^1^ post hoc test was used to analyze lesion score data. Results are shown as means ± standard error of the mean (**SEM**). The means were compared by Tukey's test (*P* < 0 .05).

## RESULTS

### Growth Performance

During the preinfection phase (1–24 d of age), different levels of PHE did not significantly affect the growth performance data (Table 3). During the infection phase (25–42 d of age) (Table 4), in the absence of the *Eimeria* challenge, growth performance was not affected by different levels of PHE (0, 200, 300, and 400 ppm) (*P* < 0.05). However, in challenged birds, ABW, DWG, and FCR linearly increased and decreased with increasing the levels of PHE, respectively (*P* < 0.05). Infected broilers had lower DFI compared to the others (*P* < 0.05).

**Table 3:** Effects of pistachio green hull aqueous extract on the performance of broilers during the starter (1–10 d) and grower phases (11–24 d).

	PHE levels (ppm)					*P* value		
Starter phase	0	200	300	400	SEM	Model	Linear	Quadratic
ABW (g/bird)	243.61	240.96	246.10	246.95	2.612	0.372	0.201	0.506
DWG (g/bird/d)	20.50	20.26	20.78	20.79	0.261	0.430	0.244	0.627
DFI (g/bird/d)	26.86	26.83	27.51	27.11	0.334	0.450	0.342	0.581
FCR (g:g)	1.32	1.34	1.33	1.32	0.009	0.414	0.652	0.130
Grower phase								
ABW (g/bird)	912.58	913.94	923.75	911.38	8.660	0.735	0.873	0.432
DWG (g/bird/d)	47.78	48.07	48.40	47.46	0.589	0.701	0.809	0.302
DFI (g/bird/d)	71.60	71.70	71.25	70.49	0.897	0.773	0.352	0.635
FCR (g:g)	1.50	1.49	1.48	1.50	0.008	0.322	0.443	0.114

Abbreviations: PHE, pistachio green hull aqueous extract; AWB, average body weight; DWG, daily weight gain; DFI, daily feed intake; FCR, feed conversion ratio; SEM, standard error of the mean.

**Table 4:** Effects of pistachio green hull aqueous extract and *Eimeria* challenge on the performance of broilers during the finisher (25–42 d) and whole phases (1–42 d).

Main effects	Finisher				Whole phase		
PHE levels (ppm)	ABW (g/bird)	DWG (g/bird/d)	DFI (g/bird/d)	FCR (g:g)	DWG (g/bird/d)	DFI (g/bird/d)	FCR (g:g)
0	2,357.65^b^	80.28^b^	133.44	1.67	55.22^b^	87.45	1.59^a^
200	2,387.29^ab^	81.85^ab^	135.28	1.66	55.93^ab^	88.26	1.58^ab^
300	2,417.73^a^	82.99^a^	134.88	1.63	56.65^a^	88.11	1.56^bc^
400	2,417.67^a^	83.68^a^	134.72	1.63	56.63^a^	87.69	1.55^c^
SEM	12.803	0.579	0.648	0.012	0.305	0.374	0.007
*Eimeria* challenge							
(-)	2,439.14^a^	84.24^a^	136.68^a^	1.63^b^	57.16^a^	88.97a	1.56^b^
(+)	2,351.03^b^	80.16^b^	132.48^b^	1.67^a^	55.06^b^	86.77b	1.58^a^
SEM	9.053	0.410	0.458	0.009	0.215	0.267	0.005
Interaction effects							
0 × (-)	2,448.88^a^	84.89^a^	136.26	1.61^b^	57.39^a^	88.82	1.55^b^
200 × (-)	2,419.14^ab^	83.26^ab^	137.25	1.64^b^	56.68^ab^	89.33	1.57^b^
300 × (-)	2,449.48^a^	84.20^a^	136.34	1.63^b^	57.41^a^	88.96	1.55^b^
400 × (-)	2,439.03^a^	84.63^a^	136.88	1.62^b^	57.14^a^	88.78	1.55^b^
0 × (+)	2,266.41^c^	75.68^c^	130.61	1.73^a^	53.04^c^	86.07	1.62^a^
200 × (+)	2,355.43^b^	80.45^b^	133.32	1.67^ab^	55.17^b^	87.20	1.58^ab^
300 × (+)	2,385.97^ab^	81.80^ab^	133.42	1.63^b^	55.89^ab^	87.25	1.56^b^
400 × (+)	2,396.31^ab^	82.73^ab^	132.57	1.63^b^	56.12^ab^	86.60	1.54^b^
SEM	18.106	0.819	0.917	0.017	0.431	0.534	0.010
*P* value							
PHE levels	0.005	0.001	0.223	0.050	0.005	0.408	0.002
*Eimeria* challenge	<.0001	<.0001	<.0001	0.005	<.0001	<.0001	0.009
PHE levels × *Eimeria* challenge	0.002	0.0001	0.525	0.006	0.002	0.807	0.0009
Linear (PHE levels at (-))	0.992	0.961	0.731	0.854	0.995	0.841	0.880
Quadratic (PHE levels at (-))	0.587	0.224	0.715	0.256	0.603	0.536	0.311
Linear (PHE levels at (+))	<.0001	<.0001	0.255	0.0002	<.0001	0.493	<.0001
Quadratic (PHE levels at (+))	0.051	0.029	0.135	0.066	0.052	0.104	0.164

Abbreviations: PHE, pistachio green hull aqueous extract; AWB, average body weight; DWG, daily weight gain; DFI, daily feed intake; FCR, feed conversion ratio; (-), unchallenged groups; (+), challenged groups; SEM, Standard error of the mean.

a,c Means within a column without a common superscript significantly differ (*P* < 0.05).

### Intestinal Morphology

The main and compound effects of PHE levels and *Eimeria* challenge on the broiler's jejunum morphology are shown in Table 5. Villus height, VH: CD ratio, and VSA decreased linearly (*P* < 0.05) with the coccidiosis challenge. However, the MT increased (*P* < 0.05) in challenged birds.

**Table 5:** Effects of pistachio green hull aqueous extract and *Eimeria* challenge on the jejunum morphology at 42 d of age.

Main effects	Jejunum morphology Features					
PHE levels (ppm)	VH (µm)	VW (µm)	CD (µm)	VH: CD	MT (µm)	VSA (mm^2^)
0	1,176.65	148.63	261.36	4.50	269.50	0.55
200	1,185.52	147.00	259.27	4.57	268.65	0.55
300	1,175.36	149.18	258.72	4.55	269.42	0.55
400	1,166.07	148.25	257.91	4.52	266.41	0.54
SEM	15.691	1.313	1.529	0.072	1.263	0.009
*Eimeria* challenge						
(-)	1,215.70^a^	149.25	259.63	4.69^a^	267.09^b^	0.57^a^
(+)	1,136.08^b^	147.21	259.00	4.39^b^	269.92^a^	0.53^b^
SEM	11.095	0.928	1.081	0.051	0.893	0.006
Interaction effects						
0 × (-)	1,221.00	149.32	260.15	4.69	266.50	0.57
200 × (-)	1,242.84	147.50	259.17	4.80	267.33	0.58
300 × (-)	1,200.15	150.50	260.15	4.62	268.67	0.57
400 × (-)	1,198.80	149.67	259.00	4.63	265.84	0.56
0 × (+)	1,132.33	148.00	262.50	4.31	272.50	0.53
200 × (+)	1,128.16	146.51	259.35	4.35	270.00	0.52
300 × (+)	1,150.51	147.49	257.32	4.48	270.16	0.53
400 × (+)	1,133.36	146.83	256.80	4.42	267.00	0.52
SEM	22.190	1.857	2.162	0.102	1.786	0.012
*P* value						
PHE levels	0.856	0.723	0.447	0.910	0.288	0.942
*Eimeria* challenge	<.0001	0.128	0.685	0.0002	0.030	<.0001
PHE levels × *Eimeria* challenge	0.493	0.928	0.623	0.419	0.521	0.844
Linear (PHE levels at (-))	0.204	0.659	0.805	0.380	0.933	0.557
Quadratic (PHE levels at (-))	0.540	0.804	0.971	0.638	0.306	0.809
Linear (PHE levels at (+))	0.825	0.746	0.055	0.404	0.059	0.966
Quadratic (PHE levels at (+))	0.799	0.810	0.530	0.660	0.857	0.923

Abbreviations: PHE, pistachio green hull aqueous extract; VH, villus height; VW, villus width; CD, crypt depth; VH: CD, villus height to crypt depth ratio; MT, muscle thickness; VSA, villus surface area; (-), unchallenged groups; (+), challenged groups; SEM, Standard error of the mean.

a,b Means within a column without a common superscript significantly differ (*P* < 0.05).

### Lesion Scoring

**Figure 1:**
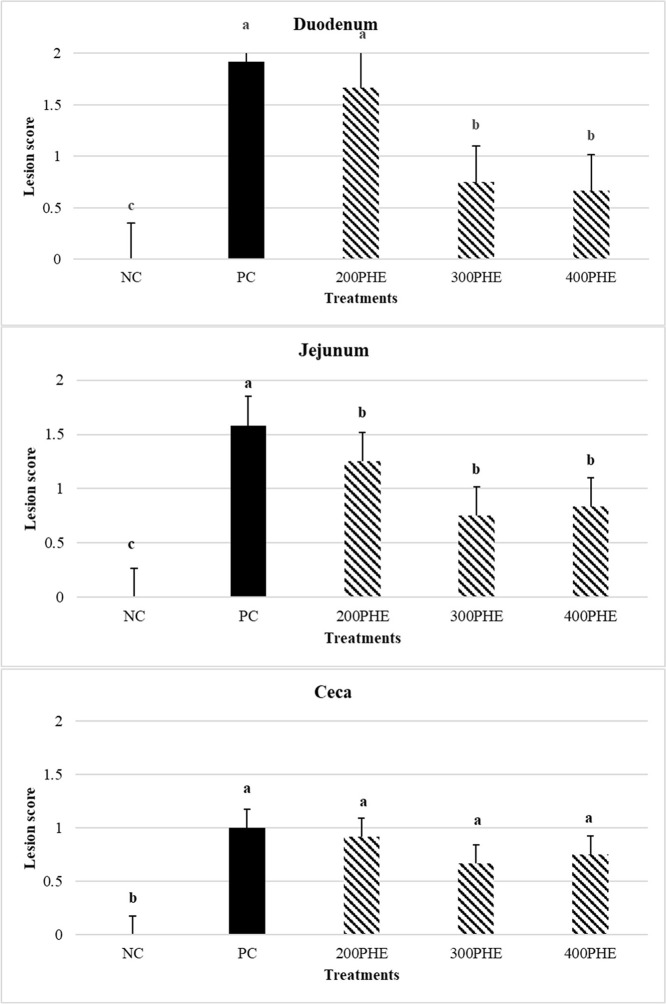
Effect of dietary supplementation of pistachio green hull extract (PHE) on lesion score (d 7 postinfection) in feces of Ross 308 male broiler chicks challenged with *Eimeria* species on d 25. NC: negative control (no challenged-no supplementation group); PC: positive control (challenged-no supplementation group); 200, 300, and 400 PHE: challenged and supplemented with 200, 300, and 400 PHE in the diet respectively). Data are presented as means ±SEM.

Figure 1 shows the main effects of PHE levels on the lesion score of duodenum, jejunum, and ceca on d 7 of post-infection. As the bar charts highlight, in the duodenum, 300 and 400 ppm of PHE considerably propitiated the lesions compared to PC, while for jejunum, in general, the use of PHE (200, 300, and 400 ppm) remarkably alleviated the lesions (*P* < 0.05). In ceca, no significant difference was observed between PHE treatments and PC (*P* > 0.05).

### Number of Oocysts Per Gram of Excreta

**Figure 2:**
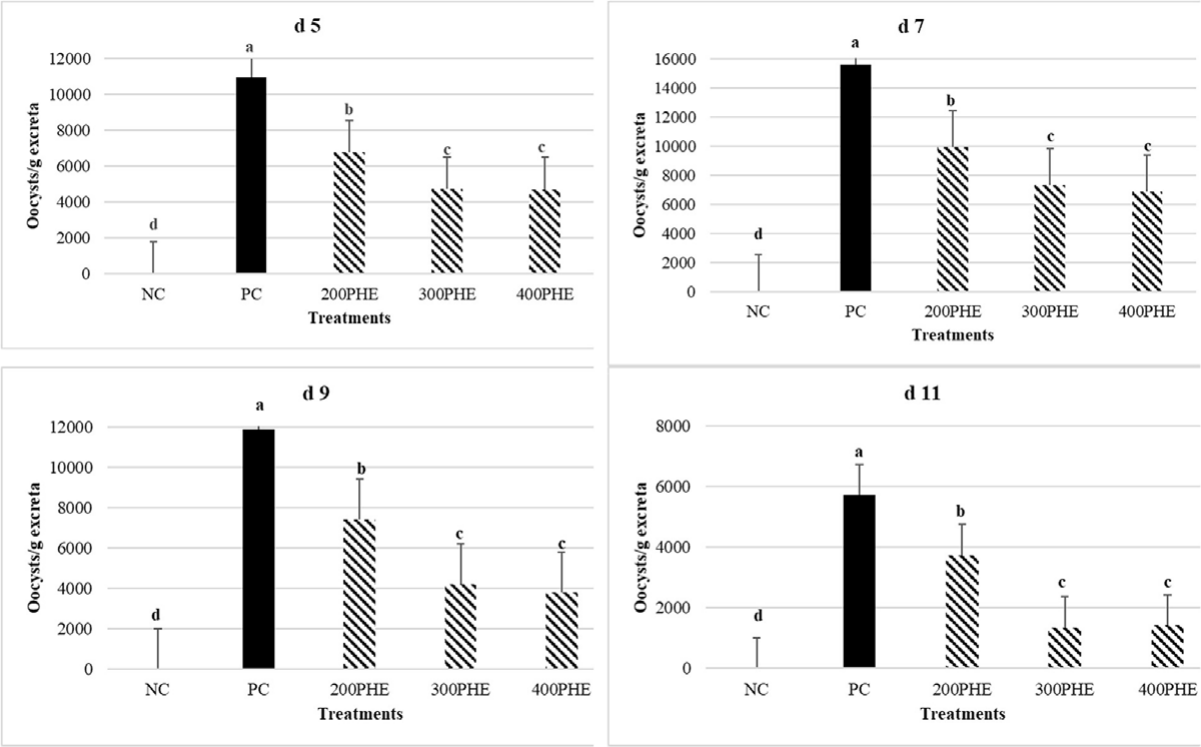
Effect of dietary supplementation of pistachio green hull extract (**PHE**) on oocyst shedding (d 5, 7, 9, and 11 postinfection) in feces of Ross 308 male broiler chicks challenged with *Eimeria* species on d 25. NC: negative control (no challenged-no supplementation group); PC: positive control (challenged-no supplementation group); 200, 300, and 400 PHE: challenged and supplemented with 200, 300, and 400 PHE in the diet respectively). Data are presented as means ±SEM.

Figure 2 indicates the effects of dietary PHE on the excreta oocyst shedding (d 5, 7, 9, and 11 postinfection). In all measurement periods, consumption of 200, 300, and 400 ppm of PHE significantly reduced the amount of oocytes compared to PC (*P* < 0.05).

### Serum antioxidant Activity

Table 6 illustrates the response of serum antioxidants to PHE levels and the *Eimeria* challenge. By increasing the levels of PHE in the diets, the amount of TAC and SOD in serum notably increased. However, birds that consumed diets containing 400 ppm PHE had the highest TAC and SOD (*P* < 0.05). The *Eimeria* challenge caused a notable reduction in TAC, SOD, and GPx levels (*P* < 0.05). Results showed that in the challenged birds, the levels of NO increased quadratically compared to healthy birds. The concentration of NO in the non-challenged and challenged birds consuming 300 and 400 ppm PHE was remarkably lower than others (*P* < 0.05).

**Table 6:** Effects of pistachio green hull aqueous extract and *Eimeria* challenge on the serum antioxidants of broilers at 42 d of age.

Main effects	Antioxidant capacity				
PHE levels (ppm)	TAC (U/mL)	NO (µmol/L)	MDA (nmol/mL)	SOD (U/mL)	GPx (U/mL)
0	18.96^b^	12.51^a^	7.28	121.83^b^	158.92
200	19.33^ab^	11.52^ab^	7.21	124.42^b^	161.17
300	19.14^ab^	10.32^b^	7.19	128.67^a^	163.67
400	19.93^a^	10.42^b^	7.24	131.20^a^	163.20
SEM	0.229	0.465	0.147	1.062	1.291
*Eimeria* challenge					
(-)	20.10^a^	9.27^b^	7.11	129.42^a^	164.83^a^
(+)	18.58^b^	13.11^a^	7.35	123.64^b^	158.64^b^
SEM	0.162	0.336	0.097	0.769	0.935
Interaction effects					
0 × (-)	19.65	9.71^b^	7.13	123.83	163.50
200 × (-)	20.14	8.29^b^	7.03	127.17	165.83
300 × (-)	19.77	9.44^b^	7.11	132.67	166.00
400 × (-)	20.84	9.66^b^	7.15	134.00	164.00
0 × (+)	18.28	15.31^a^	7.43	119.84	154.32
200 × (+)	18.52	14.75^a^	7.39	121.67	156.50
300 × (+)	18.51	11.21^b^	7.26	124.66	161.35
400 × (+)	19.03	11.19^b^	7.32	128.40	162.40
SEM	0.324	0.690	0.190	1.502	1.825
*P* value					
PHE levels	0.027	0.005	0.969	<.0001	0.053
*Eimeria* challenge	<.0001	<.0001	0.081	<.0001	<.0001
PHE levels × *Eimeria* challenge	0.835	0.005	0.939	0.615	0.129
Linear (PHE levels at [-])	0.051	0.679	0.875	<.0001	0.845
Quadratic (PHE levels at [-])	0.401	0.129	0.704	0.474	0.263
Linear (PHE levels at [+])	0.117	0.002	0.650	0.001	0.002
Quadratic (PHE levels at [+])	0.655	0.731	0.804	0.576	0.764

Abbreviations: PHE, pistachio green hull aqueous extract; TAC, total antioxidant capacity; NO, nitric oxide; MDA, malonaldehyde; SOD, superoxide dismutase; GPx, glutathione peroxidase; (-), unchallenged groups; (+), challenged groups; SEM, Standard error of the mean.

a,b Means within a column without a common superscript significantly differ (*P* < 0.05).

## DISCUSSION

There are few studies regarding the effect of PHE in broilers. Thus, the objective of this trial was to investigate the impact of different concentrations of PHE on the performance of unchallenged and *Eimeria-*challenged broilers. Previous studies reported that plants rich in antioxidant compounds such as phenols, flavonoids, tannins, and saponins can be used as alternative factors to remedy birds’ coccidiosis and have anticoccidial impacts (Hussain et al., 2021). In the present experiment, different levels of PHE had no significant effect on the ABW (finisher phase), DWG (finisher and entire experiment period), and FCR (finisher and entire experiment period) of the unchallenged birds. Noruzi and Aziz-Aliabadi (2024) also reported that using different levels of garlic (*Allium sativum*) and mushroom (*Agaricus bisporus*) powder in the broilers diet did not have any considerable effect on the growth performance. However, in the current study, in the infected birds, performance parameters increased significantly with the increase in PHE dietary levels. On the other hand, infected broilers consumed less feed compared to uninfected birds. In accordance with our results, Xu et al. (2024) attributed the improvement in growth performance and reduction in the prevalence of E. coli in chickens consuming pomegranate peel to its antioxidant properties. In the case of the non-challenged period, treatments difference was not significant. It can be said that the use of PHE is more effective when the birds are struggling with the challenge. It has been reported that pistachio green hull has significant amounts of phenolic compounds, and the high level of these compounds indicates high antioxidant and antimicrobial capabilities (Goli et al., 2005). Therefore, due to the presence of polyphenols in PHE and its antibacterial effects, it provides the basis for efficient digestion and absorption of nutrients and improves performance traits by changing the digestive tract environment (Sharifian et al., 2019). Moreover, Upadhaya et al. (2019) also reported that the *Eimeria*-infected birds fed with dietary containing essential oils showed better BWG and FCR compared to un-infected broilers without essential oils. They attributed this improvement to increased nutrient digestibility resulting from greater secretion of digestive enzymes. Khorrami et al. (2022) observed that 100 and 200 ppm of pomegranate peel extract did not have significant effect on the weight gain of *Eimeria*-challenged birds. It has been reported that thymol- and carvacrol-based encapsulated essential oils in the diet of broiler chickens under coccidiosis challenge improved body weight gain compared to the PC (challenged control) (Lee et al., 2020).

An intestine with normal operation and structure is the basis of efficient digestion and absorption of nutrients and ultimately the growth of the animal. Villus height, CD, and the VH: CD ratio are vital factors in assessing intestinal digestion and absorption in birds. The longer villus illustrates the intestinal health improvement, which in addition to a higher capacity of nutrients absorption creates uniform and integrated mucosa (Aziz-Aliabadi et al., 2023). In the current study, PHE did not have significant effect on the jejunum morphology, although *Eimeria* decreased VH, VH: CD ratio, VSA, and increased MT. According to the VSA formula, it can be said that the reason for its decrease was the reduction in the VH. It has been stated that during pathogen challenge, lymphocytes will accumulate at the site of infection and cause swelling and inflammation of the tissue and as a result increase the MT (Kaya and Tuncer, 2009). In agreement with these results, Alagbe et al. (2023) observed that the coccidia challenge reduced VH and VH: CD ratio and increased CD in the jejunum. They reported that deeper crypts and reduced VH:CD ratios in the avian gut due to *Eimeria* led to inefficient nutrient absorption due to high rate of tissue turnover rates. Jelveh et al. (2023) also stated that the coccidiosis challenge reduced VH: CD ratio. Another study (Zhang et al., 2016) showed that the coccidiosis challenge had no significant effect on the length of intestinal villi. They attributed this result to the mild to moderate level of coccidiosis.

During coccidiosis, the intestinal lining cells are infected with sporozoites, resulting in intestinal mucosa and submucosa damage (Perez-Carbajal et al., 2010). Hence, the examination of the intestinal lesion is performed to determine lesion severity. Giannenas et al. (2003) reported that the lesion scores in the *E. tenella*-inoculated group supplemented with essential oils were considerably lower than in the inoculated control group. The antimicrobial properties of herbal compounds may reduce *Eimeria* oocysts and thus reduce the damage caused by these protozoans (Giannenas et al., 2003). In the present experiment, PHE reduced lesions in the duodenum and jejunum. Khorrami et al. (2022) reported that the use of pomegranate extract decreased intestinal lesions in a dose-dependent manner. Mohiti-Asli and Ghenaatparast-Rashti (2015) also stated that the use of oregano essential oil reduced the amount of lesions in the initial and middle parts of the intestine. It has been stated that the hydroxyl substances of phenolic compounds, transport ions and protons into and out of the cell membrane. They disrupt the lipid structure of the cell membrane and enable it to penetrate ions and lead to the inhibition of enzyme activity, followed by cell death (Ultee et al., 2002). In the current study, the consumption of different levels of PHE reduced the number of oocytes excreted in the feces. The birds that received 300 and 400 ppm of PHE had the lowest amount of oocytes. However, these data could be acquired in our experiment because we induced mild infection (lesion score below 2). In line with our findings, Zhang et al. (2020) reported anticoccidial impacts of green tea extract in broiler chickens. This extract reduced the *Eimeria* infection via decreasing the mortality, oocysts numbers, and lesion score in infected broiler. Another study showed that supplementation of broilers with green tea extract considerably reduced fecal oocyst output in chickens challenged with *E. maxima*. Overall, the higher amounts of green tea showed a more protective impact and reduced fecal oocyst shedding (Jang et al., 2007). Various studies have investigated the effect of medicinal plants containing phenolic compounds such as *Aloe vera* (Desalegn and Ahmed, 2020), *Artemisia annua* (Ferreira et al., 2010), *Commiphora swynnertonii* (Bakari et al., 2012), *Curcuma longa* (Sanchez-Hernandez et al., 2019), *Emblica officinali* (Sharma et al., 2021), *Parkia biglobosa* (Ugwuoke and Pewan, 2020) and *Prunus domestica* (Muthamilselvan et al., 2016) on the birds performance and number of *Eimeria* oocytes. Their results revealed that by penetrating the coccidia oocyst wall, tannins destroy its cytoplasm and probably deactivate the endogenous enzymes responsible for the sporulation cycle in chickens. As a result, oocysts numbers will decrease.

Redox equivalence is essential to maintain cell and animal life. It refers to the free radicals production and antioxidant capacity. Total antioxidant capacity shows the total activity of all antioxidants (enzymatic or nonenzymatic) (Mao et al., 2023). It has been shown that large amounts of NO are produced by chicken monocytes and macrophages upon exposure to bacteria such as *Lactobacillus* and *Salmonella* or after *Eimeria* infection (Lee et al., 2011). Since NO is produced by immune leukocytes in response to a variety of poultry infectious pathogens, we measured serum NO levels in different treatment groups. Our outcomes showed that the concentration of serum NO was significantly higher in birds challenged with *Eimeria*. However, NO levels in broilers that consumed 300 and 400 ppm PHE were considerably reduced. This result can indicate the strong antioxidant property of PHE and its effectiveness. This finding was in line with the results of Pirali Kheirabadi et al. (2011). They concluded that *Eimeria* infection stimulated NO production, which was significant at 10 and 14 d after the challenge. In the current study, PHE significantly increased TAC and SOD levels. However, *Eimeria* notably reduced TAC, SOD, and GPx levels in the serum.Wang et al (2008a) reported an increase in serum NO concentration following the coccidiosis challenge. They stated that an increase in plasma SOD and a decrease in MDA and NO concentrations after consumption of grape seed proanthocyanidin extract indicates its antioxidant properties (Wang et al., 2008a). According to their results, herbal compounds were able to restore the oxidant-antioxidant balance that was disrupted by parasite infection (through oxidative stress) through improving the antioxidant protection system or directly eliminating free radicals (Wang et al., 2008a). It has been stated that the use of *Forsythia suspensa* extract in the broilers diet increased TAC and SOD levels and decreased MDA levels, while it had no significant effect on the GPx levels (Wang et al., 2008b). In contrast to our results, Zhang et al. (2023) did not report any significant effect of essential oil and *Eimeria* on the antioxidant capacity in birds.

## CONCLUSIONS

It can be concluded that the use of different levels of pistachio green hull aqueous extract in the broilers diets without any negative effect on the jejunum morphology, improved birds performance and antioxidant capacity. The results related to the performance, oocyte shedding and lesion scores emphasized that the pistachio green hull aqueous extract will perform more efficiently in the presence of *Eimeria* challenge. It is recommended to use 300 to 400 ppm of green hull aqueous in the broilers diet within coccidiosis infection.

## DISCLOSURES

The authors declare no conflicts of interest.

## Footnotes

1 Dwass-Steel-Critchlow-Fligner.
